# Drastic alterations in the loop structure around colchicine upon complex formation with an engineered lipocalin indicate a conformational selection mechanism

**DOI:** 10.1107/S2053230X23006817

**Published:** 2023-08-16

**Authors:** Elena Jerschke, Andreas Eichinger, Arne Skerra

**Affiliations:** a Lehrstuhl für Biologische Chemie, Technische Universität München, Emil-Erlenmeyer-Forum 5, 85354 Freising, Germany; Osaka University, Japan

**Keywords:** Anticalins, hapten recognition, induced fit, protein design

## Abstract

Using Anticalin technology, a lipocalin protein dubbed Colchicalin, with the ability to bind the toxic plant alkaloid colchicine with picomolar affinity, has previously been engineered, thus offering a potential antidote *in vivo* and also allowing its sensitive detection in biological samples. To further analyze the mode of ligand recognition, the crystal structure of Colchicalin is now reported in its unliganded form and is compared with the colchicine complex. A superposition of the protein structures revealed major rearrangements in the four structurally variable loops of the engineered lipocalin. Notably, the binding pocket in the unbound protein is largely occupied by the inward-bent loop #3, in particular Ile97, as well as by the phenylalanine side chain at position 71 in loop #2. Upon binding of colchicine, a dramatic shift of loop #3 by up to 11.1 Å occurs, in combination with a side-chain flip of Phe71, thus liberating the necessary space within the ligand pocket. Interestingly, the proline residue at the neighboring position 72, which arose during the combinatorial engineering of Colchicalin, remained in a *cis* configuration in both structures. These findings provide a striking example of a conformational adaptation mechanism, which is a long-known phenomenon for antibodies in immunochemistry, during the recognition of a small ligand by an engineered lipocalin, thus illustrating the general similarity between the mode of antigen/ligand binding by immuno­globulins and lipocalins.

## Abstract 2

Colchicalin is an engineered binding protein derived from the lipocalin scaffold, which provides a binding site comprised of four loops exhibiting structural plasticity. Structural analysis of this lipocalin in the absence and the presence of its ligand colchicine reveals drastic structural changes which indicate a conformational selection mechanism.

## 1. Introduction

Anticalin proteins are derived from natural lipocalins via combinatorial protein design and directed evolution to recognize a variety of medically relevant target molecules, thus offering prospects for theranostic applications (Deuschle *et al.*, 2021 ▸; Rothe & Skerra, 2018 ▸). With as low as 10% mutual amino-acid sequence identity, the natural members of the lipocalin protein family exhibit a highly conserved cup-shaped fold which is dominated by a central β-barrel made of eight antiparallel β-strands with an adjacent α-helix (Skerra, 2000 ▸). An extended ligand pocket is shaped by four structurally variable loops at the open end of the β-barrel, which leads to diverse ligand-binding activities for the natural lipocalins (Breustedt *et al.*, 2006 ▸; Schiefner & Skerra, 2015 ▸). In connection with methods of targeted random mutagenesis and molecular selection, this molecular architecture enables the reshaping of the lipocalin binding site to tightly bind a wide range of prescribed molecular targets from small molecules through peptides to proteins (Richter *et al.*, 2014 ▸). In fact, the mode of ligand binding by the resulting Anticalin proteins resembles the well known recognition of antigens via the six complementarity-determining regions (CDRs) of antibodies (Skerra, 2003 ▸; Achatz *et al.*, 2022 ▸).

Colchicalin was developed against the plant poison colchicine starting from human lipocalin 2 (Lcn2; Gebauer & Skerra, 2012 ▸; Barkovskiy *et al.*, 2019 ▸). After selection from a naïve random library and several cycles of affinity maturation, the Anticalin D6.2 (the V69M/I80T/F83L triple mutant of the initial hit D6.1) revealed a remarkably low *K*_d_ value of 120 p*M*. The most significant mutation during this maturation process, Val69 to Met, led to a tenfold increase in affinity, but at the expense of higher oxidation sensitivity due to the introduction of a surface-exposed methionine residue (Teh *et al.*, 1987 ▸). Consequently, Met69 was replaced by the chemically more robust and hydrophilic glutamine side chain with a similar size. The resulting variant, D6.3, retained a picomolar affinity towards colchicine. An N-terminally truncated version of D6.3, Δ4-D6.3 (also devoid of an affinity tag), was crystallized in complex with colchicine (Barkovskiy *et al.*, 2019 ▸). Its X-ray structural analysis confirmed the canonical molecular architecture of the lipocalin fold, with the deeply embedded ligand in the central cavity at the open end of the β-barrel. While major structural rearrangements of the variable loop region were observed in comparison to the wild-type Lcn2 scaffold (Barkovskiy *et al.*, 2019 ▸), including a unique *cis*-proline bond between Phe71 and Pro72, it was unclear whether these changes were a consequence of the amino-acid exchanges that had been introduced into the loop region of the protein scaffold or were driven by complex formation with the ligand.

Whereas the antigen-binding sites of antibodies (immuno­globulins, Igs) can exist in a preformed high-affinity conformation towards their molecular target and associate with it via a rigid lock-and-key mechanism (Al Qaraghuli *et al.*, 2021 ▸), an ability to structurally adapt to the cognate ligand has been elucidated in several instances (Al Qaraghuli *et al.*, 2020 ▸, 2021 ▸; Stanfield & Wilson, 1994 ▸). The corresponding rearrangements extend from local changes within CDRs that directly interact with the antigen to more complex alterations in quaternary structure that affect the angle of V_H_/V_L_ pairing and may even propagate further into the constant Ig region (Rini *et al.*, 1992 ▸; Al Qaraghuli *et al.*, 2020 ▸, 2021 ▸; Stanfield & Wilson, 1994 ▸).

Such spatial shifts in the hypervariable loops of the antibody in response to antigen binding can be described by two alternative mechanisms: (i) induced fit and (ii) conformational selection. In the case of induced fit, initial contact formation with the antigen is followed by conformational rearrangement of the binding site, which eventually results in tight complex formation (Stanfield & Wilson, 1994 ▸; Rini *et al.*, 1992 ▸; Berger *et al.*, 1999 ▸). On the other hand, conformational selection depends on the simultaneous population of both high- and low-affinity conformations of the ligand-free state of the antibody (Rini *et al.*, 1992 ▸; Berger *et al.*, 1999 ▸); thus, the target molecule can engage with the fraction of the antibody in the structurally more competent conformation, even though its proportion may be low. This results in a shift of the (bio)chemical equilibrium between the (at least) two different conformations according to the principle of Le Chatelier. However, in the majority of practical instances it has to be assumed that a combination of both mechanisms contributes to antigen recognition (Berger *et al.*, 1999 ▸).

Notably, structural rearrangements in response to ligand binding have been reported for several Anticalins where X-ray structures have been elucidated for both the complex and the free state (Schönfeld *et al.*, 2009 ▸; Kim *et al.*, 2009 ▸; Dauner *et al.*, 2018 ▸). In these cases a simple induced-fit mechanism was sufficient to describe the observed structural change, as an open pocket was observed in the unbound state, thus offering unhindered access by the ligand, followed by structural adaptation. Here, we report the three-dimensional structure of Colchicalin D6.2 in its ligand-free state, which unexpectedly reveals a closed binding site, indicating the need for considerable conformational rearrangement prior to complex formation with colchicine.

## 2. Materials and methods

### 2.1. Preparation of Colchicalin D6.2 for X-ray analysis

Colchicalin D6.2 was produced with a C-terminal His_6_ tag in the less reducing cytoplasm of *Escherichia coli* Origami B (EMD Millipore, Billerica, Massachusetts, USA) using the vector pASK75-T7RBS2-D6.2-His_6_, which is a derivative of the generic vector pASK75 (Skerra, 1994 ▸). To this end, the signal peptide was replaced by a start methionine residue; otherwise, the encoded protein corresponded to the full-length mature Anticalin protein as described previously (Barkovskiy *et al.*, 2019 ▸). A 2 l shake-flask culture was grown at 37°C using TB medium supplemented with 100 mg l^−1^ ampicillin until an OD_550_ of 0.5 was reached. The temperature was then lowered to 26°C and recombinant gene expression was induced at an OD_550_ of 1.0 with 200 ng ml^−1^ anhydro­tetra­cycline (Acros Organics, Geel, Belgium). After 15 h of further incubation, the bacteria were harvested by centrifugation at an OD_550_ of ∼10.5. After resuspension of the bacterial pellet in 100 ml 40 m*M* HEPES–NaOH pH 7.5, 500 m*M* NaCl, the total cell extract was prepared from two cultures by mechanical lysis using a PandaPLUS 2000 cell-disruption system (GEA Niro Soavi, Lübeck, Germany). The lipocalin variant was then purified by immobilized metal ion-affinity chromatography (IMAC) on a 25 ml Ni-Sepharose High Performance column (GE Healthcare, Freiburg, Germany) using an ÄKTApurifier system (GE Healthcare) with 40 m*M* HEPES–NaOH pH 7.5, 500 m*M* NaCl as the running buffer and a concentration gradient of 0–500 m*M* imidazole–HCl. Elution fractions containing D6.2-His_6_ were immediately supplemented with Na EDTA pH 8.0 to a final concentration of 10 m*M* to prevent metal ion-induced protein precipitation. The resulting protein solution was then dialyzed against 20 m*M* MES–NaOH pH 6.0 and further purified by cation-exchange chromatography via two repeated runs on a 6 ml Resource S column (GE Healthcare). The purity of the final sample was assessed by 15%(*w*/*v*) SDS–PAGE with Coomassie Brilliant Blue staining as well as by mass spectrometry on a maXis ESI-QTOF instrument (Bruker Daltonics, Bremen, Germany) in positive-ion mode. Prior to protein crystallization, the sample was finally dialyzed against 10 m*M* HEPES–NaOH pH 7.5, 50 m*M* NaCl and then concentrated to 45.8 mg ml^−1^ by ultrafiltration using an Amicon Ultra-4 10 kDa cutoff concentrator (Merck Millipore, Darmstadt, Germany) and sterile-filtered with an Ultrafree-MC GV centrifugal filter (PVDF, 0.22 µm; Merck Millipore).

### 2.2. Colchicine affinity measurements

To assess the influence of Ca^2+^ on the affinity of D6.2 towards colchicine, the equilibrium dissociation constant (*K*_d_) was measured by fluorescence titration using an LS50B spectrophotometer (Perkin Elmer, Waltham, Massachusetts, USA) as described previously (Barkovskiy *et al.*, 2019 ▸). In brief, 2 ml purified D6.2-His_6_ at 100 n*M* in 10 m*M* HEPES–NaOH pH 7.5, 50 m*M* NaCl with or without the addition of 200 m*M* CaCl_2_ was equilibrated in a 4 ml quartz cuvette (Hellma Analytics, Müllheim, Germany) for 10 min at ambient temperature. A stock solution of 40 µ*M* colchicine (Applichem, Darmstadt, Germany) in the same buffer was then added stepwise in 0.25–4 µl aliquots, followed by stirring for 60 s. The tyrosine and tryptophan fluorescence (excitation at 280 nm, emission at 345 nm) was measured at each colchicine concentration for 15 s under stirring. The inner filter effect, due to the absorption of colchicine at 345 nm, was corrected by performing a control titration of *N*-acetyltryptophanamide as described by Vopel *et al.* (2005 ▸). The resulting data were fitted according to bimolecular complex formation (Barkovskiy *et al.*, 2019 ▸) with *Origin Pro* (Origin­Lab, Northampton, Massachusetts, USA).

### 2.3. Protein crystallization and structure solution

For X-ray structure determination, the purified Colchicalin D6.2 solution obtained as described above was subjected to an in-house crystallization screen using the vapor-diffusion technique. A single crystal was obtained at 20°C using a sitting drop prepared from 300 nl purified protein solution (concentrated to 45.8 mg ml^−1^; see above) and 300 nl precipitant solution consisting of 20%(*w*/*v*) PEG 3350, 200 m*M* CaCl_2_. The crystal was cooled in liquid nitrogen after adding 25%(*v*/*v*) glycerol as a cryoprotectant, and a single-wavelength X-ray diffraction data set was collected at 100 K on BESSY beamline 14.1, Helmholtz-Zentrum Berlin, Germany. The data were processed and the crystal structure was solved utilizing the coordinates of the Colchicalin–colchicine complex (PDB entry 5nkn) as a search model for molecular replacement, followed by refinement using the *CCP*4 suite (Agirre *et al.*, 2023 ▸), as described previously (Barkovskiy *et al.*, 2019 ▸). Average *B*-factor values were calculated with *BAVERAGE* from *CCP*4. In a separate theoretical study, protein modeling on the basis of the amino-acid sequence of Colchicalin (Barkovskiy *et al.*, 2019 ▸) was performed using the *AlphaFold*2.0 system (Jumper *et al.*, 2021 ▸) running on a local desktop computer (Max template date 30 March 2017). The atomic coordinates and structure factors of the Colchicalin crystal structure in the uncomplexed state have been deposited in the Protein Data Bank (PDB), Research Collaboratory for Structural Bio­informatics, Rutgers University, New Brunswick, New Jersey, USA under accession code 6z6z.

## 3. Results

**Figure 1:**
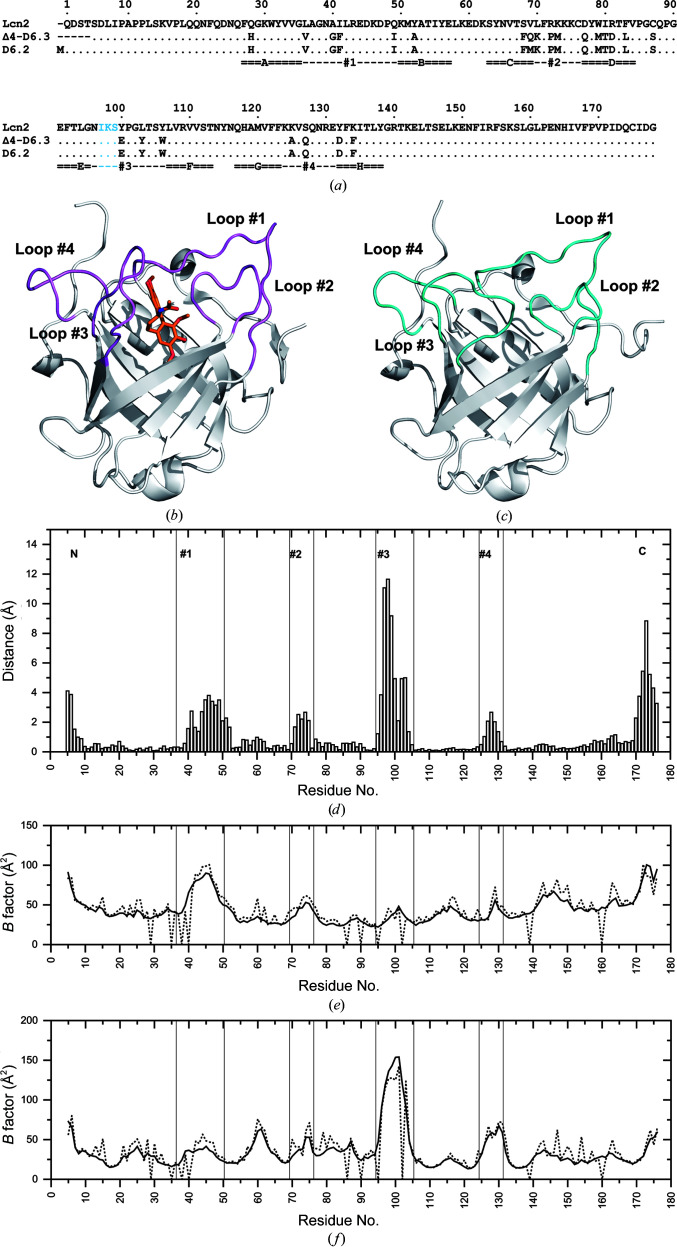
Comparison of Colchicalin in the presence or absence of the ligand colchicine. (*a*) Amino-acid sequence alignment of Colchicalin D6.2, crystallized in this study without ligand, with the previously crystallized Colchicalin Δ4-D6.3 in complex with colchicine (Barkovskiy *et al.*, 2019 ▸) as well as wild-type Lcn2. Positions Ile97, Lys98 and Ser99 on loop #3, which do not directly contact the ligand, exhibit the largest deviation between the apo and complexed states of Colchicalin and are marked in blue. (*b*) Overall architecture of the previously described Colchicalin Δ4-D6.3 in complex with colchicine (orange). (*c*) Overall architecture of Colchicalin D6.2 in its apo state. The marked conformational rearrangement of loop #3 is evident (loops colored magenta and cyan, respectively). (*d*) Spatial deviation between C^α^ positions in the crystal structure of apo Colchicalin *versus* the Colchicalin–colchicine complex after superposition via the 58 conserved C^α^ atoms in the lipocalin fold (Skerra, 2000 ▸). The structurally variable loop regions 1–4 are indicated. Note the high values for loop #3. (*e*, *f*) *B*-factor plots for the two Colchicalin crystal structures. The average *B*-factor values are shown separately for the main-chain atoms (solid lines) and side-chain atoms (broken lines) of each residue in the apoprotein (*e*) and the Colchicalin–colchicine complex (*f*).

The crystal structure of Colchicalin D6.2 in the absence of its cognate ligand was solved at a resolution of 1.8 Å by molecular replacement using the previously published coordinate set of the Colchicalin–colchicine complex (Barkovskiy *et al.*, 2019 ▸; Table 1 ▸). The refined structural model, with one protein molecule in the asymmetric unit in space group *I*222, comprises residues Ser5–Ile176 with continuous electron density. To compare the structure of the D6.2 apoprotein with the previously described crystal structure of Colchicalin Δ4-D6.3 in complex with colchicine (Barkovskiy *et al.*, 2019 ▸) and to study the mechanism of ligand binding, the coordinate sets were superimposed (Figs. 1 ▸ and 2 ▸). In accordance with previous investigations of other engineered lipocalins (Richter *et al.*, 2014 ▸; Achatz *et al.*, 2022 ▸), the β-barrel fold remained fully preserved irrespective of the presence of colchicine, with a root-mean-square deviation (r.m.s.d.) of 0.4 Å for the 58 C^α^ atoms of the β-barrel that are structurally conserved across the lipocalin protein family (Skerra, 2000 ▸).

**Figure 2:**
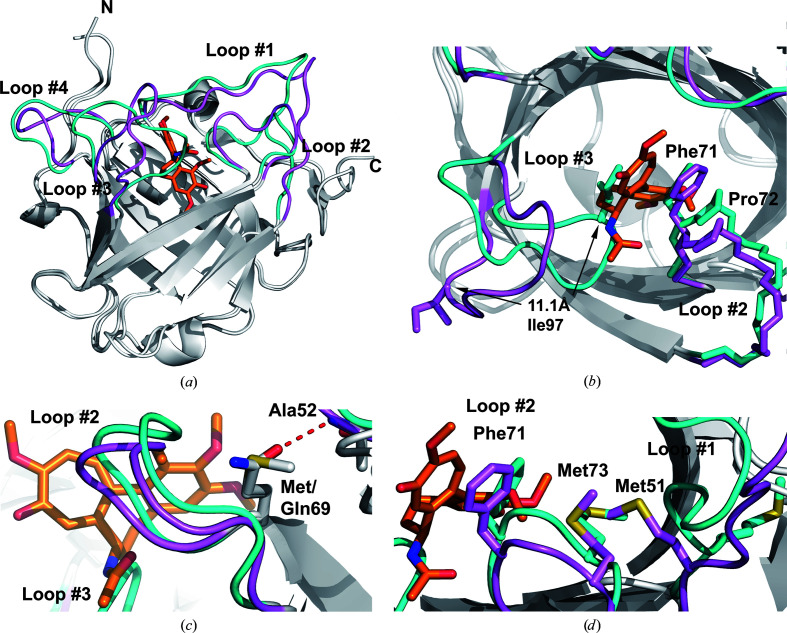
X-ray crystallographic analysis of Colchicalin in the apo form compared with its complex with colchicine. (*a*) Superposition of Colchicalin D6.2 in the apo state (loops in cyan) with Colchicalin Δ4-D6.3 in complex with colchicine (PDB entry 5nkn; loops in violet). (*b*) Binding mechanism for colchicine. In the apo state the side chains of Ile97 in loop #3 and of Phe71 in loop #2 point into the ligand pocket (cyan). In the complex, the drastic rearrangement of loop #3 in combination with the (upward/outward) side-chain flip of Phe71 enables the tight fit of colchicine within the ligand pocket. Of note, the *cis*-peptide bond between Phe71 and Pro72 in loop #2 is present in both structures. (*c*) Side-chain conformations of Met69 in the apo structure in comparison with Gln69 in the structure of the ligand complex. The carboxamide group of glutamine forms an additional hydrogen bond to the main-chain O atom of Ala52 (red dashed line). (*d*) A concerted change in side-chain conformations upon colchicine binding. In the complex, the side chains of Met51 at the C-terminal end of loop #1 and Met73 in loop #2 (violet) are directed towards the bound colchicine, whereas in the apo state they point away from the binding site. It seems that the inward movement of the Phe71 side chain in the apoprotein drags along the neighboring residue Met73 in loop #2, whose bulky side chain laterally pushes aside that of Met51 in loop #1.

However, a much higher overall r.m.s.d. of 2.1 Å was calculated upon pairwise superposition of all 172 C^α^ atoms (Ser5–Ile176), which was mainly due to the markedly different conformations of the four loops that form the binding site (Fig. 1 ▸ *d*). The size of the ligand pocket in the apoprotein is 17 × 17 Å (measured between the C^α^ atoms of the residue pairs Gly40/Pro101 and Phe71/Arg130, respectively) compared with dimensions of 15 × 20 Å in the complex between Colchicalin and colchicine. The side-chain conformation of Met69 in D6.2 on the surface of the β-barrel, the only differing amino acid between the structures (apart from the four N-terminally deleted residues in Δ4-D6.3 and the unresolved start methionine residue in D6.2), is very similar to that of its counterpart glutamine in the complex of Δ4-D6.3 with colchicine. In the latter structure an additional hydrogen bond can be seen between the side chain of Gln69 and the backbone O atom of Ala52 (Fig. 2 ▸ *c*).

Regarding the complex formation with colchicine, structural effects on the ligand pocket of the engineered lipocalin are evident for the side chains of Met51, Phe71 and Met73 (Table 2 ▸). These are directed away from the binding site in the apo state, whereas in the complex they point towards the bound colchicine (Fig. 2 ▸ *d*). This concerted side-chain re­orientation is accompanied by conformational changes in all four structurally variable loops at the entrance to the ligand pocket. For example, in the apo state loop #1 is slightly bent towards the binding pocket, with a maximum deviation of 3.5 Å at Ile49 (C^α^ atom). Notable structural deviations are also seen for loop #4, the N-terminal part of which, up to Gln128, is bent outwards by up to 3.7 Å, whereas the following segment is directed inwards by up to 3.6 Å at Arg130 (C^α^ atom). Interestingly, while the side chain of Phe71 is buried in the pocket of the apoprotein and swings out in the ligand complex, the *cis*-peptide configuration of the peptide bond to the following residue, Pro72, in loop #2 remains preserved between both states.

Remarkably, the largest deviation occurs at Gly95–Ser105 in loop #3, which is bent inwards in the apo­protein by up to 11.1 Å at the C^α^ atom of Lys98 (which is not in contact with the bound ligand) in both states after superposition via the set of 58 conserved C^α^ atoms (see Figs. 1 ▸ *b* and 2 ▸ *b*). As a result, the side chain of Ile97 occupies the ligand pocket together with the Phe71 side chain in its altered conformation as described above. While Phe71 rests in a position that is otherwise filled by the trimethoxyphenylene moiety (ring A), Ile97 replaces the aminocyclohexyl ring (B) of the bound colchicine in the complex. Conversely, loop #3 is bent outwards in the colchicine complex, thus providing space for the ligand. Calculation of the ligand-pocket volumes using *CASTp* (Tian *et al.*, 2018 ▸) led to 343 Å^3^ for the Colchicalin–colchicine complex (after removal of the ligand) compared with a much smaller value of 63 Å^3^ for the apo state, which clearly demonstrates the functional effect of the change in loop conformation.

The fact that loop #3 gives rise to this kind of steric hindrance for colchicine binding in the present crystal structure suggests the presence of at least one additional conformation with an accessible binding pocket in the apo state of Colchicalin, presumably in a low proportion in solution, in order to provide a mechanism for complex formation with colchicine. Interestingly, a three-dimensional structure prediction based on the amino-acid sequence of Colchicalin using the *AlphaFold*2.0 system (Jumper *et al.*, 2021 ▸) did not provide any indication of alternative loop conformations in this engineered lipocalin. In both cases, all five most highly ranked models resembled the loop conformations of the wild-type Lcn2 protein, the crystal structure of which differs considerably from Colchicalin both in the apo and the holo state in all loops except for loop #1 (Achatz *et al.*, 2022 ▸).

A potential influence of crystal packing on the conformation of loop #3 in the structure of the apo state can be excluded as the entire loop resides in a solvent-filled channel within the crystal. Nevertheless, the backbone is well defined in the electron density and the entire loop shows low *B* factors (Fig. 1 ▸ *e*). On the other hand, residues 97–103 at the tip of the loop are poorly defined in the crystal structure of the Colchicalin–colchicine complex, which is reflected by high local *B* factors (Fig. 1 ▸ *f*) and could indicate multiple populated conformational states in this region. However, without doubt Ile97 points away from the binding pocket, which is fully occupied with the ligand in the complex.

Of note, during refinement of the crystal structure additional 2*F*_o_ − *F*_c_ electron density with a spherical shape appeared near loop #3 of apo Colchicalin. Considering that the precipitant solution contained 200 m*M* CaCl_2_, a Ca^2+^ ion was modeled at this position (*B* factor 25.3 Å^2^), leading to an octahedral coordination sphere involving Gly95 (main-chain carbonyl), Asn96 (side-chain carboxamide), Glu100 (side-chain carboxylate) and Thr104 (main-chain carbonyl) as well as two water molecules. However, a pentagonal bipyramidal coordination is preferred in natural calcium-binding sites and often more than one side-chain carboxylate groups are found as ligands (sometimes in a bidentate fashion; Kirberger *et al.*, 2008 ▸). Still, the question arose whether binding of the calcium ion in the crystallized protein may play a role in the new conformation of loop #3.

**Figure 3:**
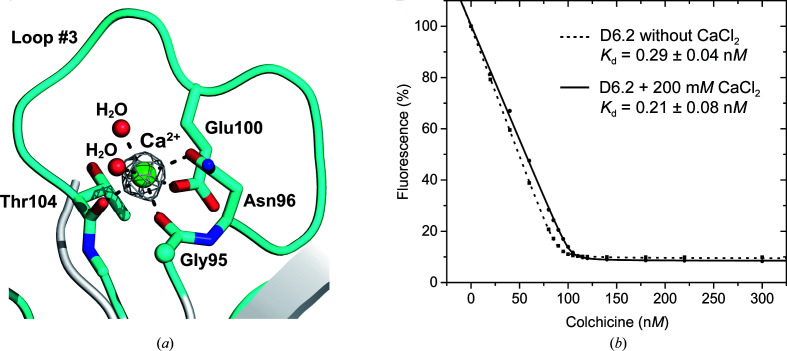
The role of a bound Ca^2+^ ion in the crystal structure of ligand-free Colchicalin. (*a*) A Ca^2+^-binding site with octahedral coordination in the crystal structure of the apoprotein. The four residues that contact the metal ion, two side chains and two main-chain carbonyl O atoms, as well as two coordinating water molecules, are depicted as sticks or small spheres. The 2*F*_o_ − *F*_c_ electron density around the Ca^2+^ ion is shown contoured at 5σ. (*b*) Fluorescence titration of Colchicalin D6.2 with colchicine in the presence or absence of 200 m*M* CaCl_2_ reveals no significant influence on the ligand affinity.

In fact, such a metal-induced conformational change should strongly influence the affinity towards colchicine as loop #3 harbors several ligand-contacting residues: Glu100, Pro101, Gly102 and Tyr103 (Fig. 3 ▸). Thus, we measured the colchicine-binding activity of the D6.2-His_6_ protein by fluorescence titration in the presence or absence of 200 m*M* CaCl_2_. As a result (Fig. 3 ▸ *b*), there was no detectable effect on the *K*_d_ value for colchicine, even at such a high concentration of calcium. Therefore, coordination of the calcium ion is most likely to be an accidental consequence of crystal packing (which occurs in space group *I*222 for apo Colchicalin compared with *P*4_1_22 for the Colchicalin–colchicine complex) and is functionally irrelevant.

## 4. Discussion

Prior to this study, evidence for structural adaptation of an engineered lipocalin loop region to the bound target has only been observed in a few cases when investigating X-ray structures of both ligand-free Anticalins and their complexes (Kim *et al.*, 2009 ▸; Dauner *et al.*, 2018 ▸; Schönfeld *et al.*, 2009 ▸). For instance, comparison of the unbound Anticalin Tb7.14 (PDB entry 3dtq) with the closely related mutant Tb7N9 in complex with Y^3+^-DTPA (PDB entry 3dsz) revealed only minor shifts of loops #2, #3 and #4, with the largest deviation of 1.3 Å seen for Gly102 in loop #3. Loop #1 displayed considerable backbone plasticity but was not involved in ligand contacts (Kim *et al.*, 2009 ▸). In another structural analysis, a comparison between Petrocalin with the bound ligand petrobactin and the ligand-free lipocalin (PDB entries 6gqz and 6gr0, respectively) indicated larger rearrangements within the loop region. Loop #2 moved 4.3 Å and loop #3 moved 2.0 Å towards the bound ligand (measured at the C^α^ atoms of Glu74 and Ser99 of chain *A*, respectively), thus providing evidence for an induced fit upon complex formation (Dauner *et al.*, 2018 ▸). Finally, in the case of the Anticalin PRS-050, which binds the protein target CTLA-4, significant conformational rearrangements of loops #2, #3 and #4 were measured as result of complex formation (PDB entries 3bx7 and 3bx8, respectively). In fact, a pronounced induced fit was observed for loops #3 and #4, which changed their conformations from disordered to ordered in the corresponding X-ray structures (Schönfeld *et al.*, 2009 ▸).

**Figure 4:**
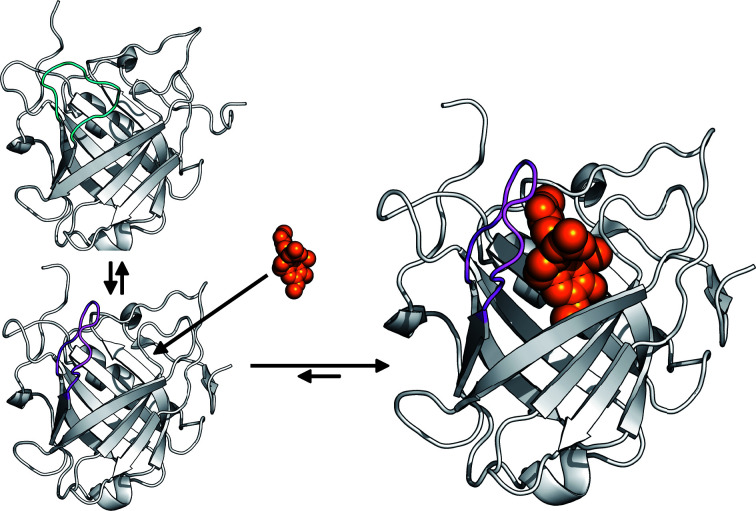
Schematic illustration of the principle of conformational selection upon the binding of colchicine to Colchicalin. Prior to complex formation, Colchicalin exists in solution in at least two states, one with an open and one with an occluded ligand pocket, and only the open state can accommodate colchicine.

Compared with these previous examples, the movement of loop #3 by ∼11 Å in the Colchicalin structure as a result of ligand binding is by far the largest structural change. Remarkably, the binding site is occupied by Ile97, together with Phe71 in loop #2, in the apo state and loop #3 has to move outwards from the ligand pocket in order for colchicine to find its place. Hence, an ‘induced fit’ in the conventional sense, where a target complex is initially formed and then triggers conformational changes, cannot apply. Instead, it is more plausible to assume that both observed conformations of loop #3 exist in the apo state, with the open conformation probably less occupied but still sufficiently available for complex formation with colchicine, which is accompanied by a shift in the equilibrium (Fig. 4 ▸). This kind of mechanism would be in accordance with the concept of ‘conformational selection’ (Berger *et al.*, 1999 ▸). Whether loop #3 may exhibit additional conformations in solution, or even might be fully mobile, remains to be studied, for example using NMR analysis.

It has previously been recognized that the binding mode of both natural and engineered lipocalins to their target molecules resembles the interaction between antigens and immunoglobulins (Skerra, 2003 ▸). In the case of antibodies, six hypervariable loops (also known as complementarity-determining regions, CDRs) are mounted on a conserved β-sandwich framework and govern the specific and tight complex formation with the antigen. This molecular architecture is comparable to the lipocalins, where the four structurally variable loops that form the cup-shaped binding site are supported by a rigid β-barrel.

For antibodies, two distinct modes of target binding have been observed: (i) a rigid lock-and-key interaction and (ii) varying degrees of conformational rearrangement upon complex formation. As mentioned further above, in principle two mechanisms of spatial adaptation have been recognized: (i) induced fit, in which conformational alteration follows ligand binding, and (ii) conformational selection, which is based on a pre-existing mixture of conformational states (Rini *et al.*, 1992 ▸; Berger *et al.*, 1999 ▸).

To date, studies of engineered lipocalins have provided evidence for induced fit upon ligand binding, including significant backbone rearrangements of the loop region towards the bound target molecule that were accompanied by shifts and rotations of individual amino-acid side chains (Schönfeld *et al.*, 2009 ▸; Kim *et al.*, 2009 ▸; Dauner *et al.*, 2018 ▸). In the present study, we observed the largest shift to date of a structurally variable loop (loop #3) associated with ligand binding in the case of an Anticalin selected against colchicine. Together with a side-chain flip of Phe71 by about 120°, and a few less drastic side-chain alterations, this movement, starting from the apo state, creates a perfectly complementary pocket to the tricyclic colchicine ligand, whereas the β-barrel scaffold remains structurally unchanged. Conversely, due to the peculiar conformations of loop #3 and Phe71 in the unbound state, colchicine is sterically hindered from forming an initial complex with Colchicalin.

Therefore, it is reasonable to assume that the open conformation must also exist in the absence of the ligand, possibly with a scarce population, such that the binding site is fundamentally accessible. Hence, the mechanism of conformational selection appears to describe the mode of binding for this Anticalin more accurately (Fig. 4 ▸). While it cannot be fully excluded that the structure of the binding site in either the apo or the holo state of Colchicalin, as evident from the X-ray analyses, is influenced by the crystal environment (such as the bound calcium ion from the precipitant solution or direct/indirect packing contacts with a neighboring molecule), the manner in which the side chains of Phe71 and Ile97 occupy the same pocket in the apo state where the ligand colchicine is bound in the complex is compelling.

Taken together with the previous studies mentioned above, the case of the Colchicalin–colchicine pair with its distinct protein conformations in the presence and absence of the ligand provides another example that nicely illustrates the structural and functional analogy between (engineered) lipocalins and antibodies with regard to the mechanisms of ligand/antigen recognition.

## Supplementary Material

PDB reference: an Anticalin directed towards colchicine without a ligand, 6z6z

## Figures and Tables

**Table 1:** Crystallographic data and refinement statistics Values in parentheses are for the highest resolution shell.

Data collection	
Space group	*I*222
*a*, *b*, *c* (Å)	38.6, 75.6, 116.1
α, β, γ (°)	90.0, 90.0, 90.0
Resolution range (Å)	63.34–1.78 (1.88–1.78)
〈*I*/σ(*I*)〉	6.5 (1.8)
*R*_merge_ (%)	6.0 (41.8)
Observed/unique reflections	175641/16656
Completeness (%)	99.6 (99.8)
Refinement statistics	
*R*_cryst_/*R*_free_ (%)	20.1/25.9
No. of protein atoms	1404
No. of water molecules	61
Average *B* factor (Å^2^)	
Protein	45.4
Solvent	39.1
Ramachandran analysis†	
Core (%)	86.5
Allowed (%)	12.8
Generously allowed (%)	0.0
Disallowed (%)	0.7

† Calculated with *PROCHECK* (Laskowski *et al.*, 1993 ▸).

**Table 2:** Structural alterations in apo Colchicalin upon binding colchicine

Residue	C^α^ deviation† (Å)	Region
Val33	0.4	β-Strand A
Val36	0.3	β-Strand A
Gly40	1.4	Loop #1
Phe41	2.7	Loop #1
Met51	2.3	Loop #1
Thr54	0.3	β-Strand B
Tyr56	0.7	β-Strand B
Val66	0.5	β-Strand C
Phe68	0.3	β-Strand C
Met/Gln69	0.2	β-Strand C
Lys70	1.8	Loop #2
Phe71	1.7	Loop #2
Met73	2.2	Loop #2
Met79	0.4	β-Strand D
Asp81	0.6	β-Strand D
Leu94	0.2	β-Strand E
Gly102	4.8	Loop #3
Thr104	1.4	Loop #3
Trp106	0.1	β-Strand F
Phe123	0.2	β-Strand G
Arg130	3.6	Loop #4
Asp132	0.3	Loop #4
Phe134	0.2	β-Strand H
Thr136	0.3	β-Strand H
Tyr138	0.4	β-Strand H

† Distances between C^α^ atoms in the apoprotein (PDB entry 6z6z) and Colchicalin in complex with colchicine (PDB entry 5nkn) for those residues which form contacts to the bound ligand after superposition of both crystal structures via the 58 conserved C^α^ positions in the lipocalin fold (Skerra, 2000 ▸).
